# Supplementary figures and images for: Constraining models of dominance for nonsynonymous mutations in the human genome
Source: PLoS Genet. 2024 Sep 20;20(9):e1011198. doi: 10.1371/journal.pgen.1011198 (PMC11446423; doi:10.1371/journal.pgen.1011198)

## SFS for gamma model

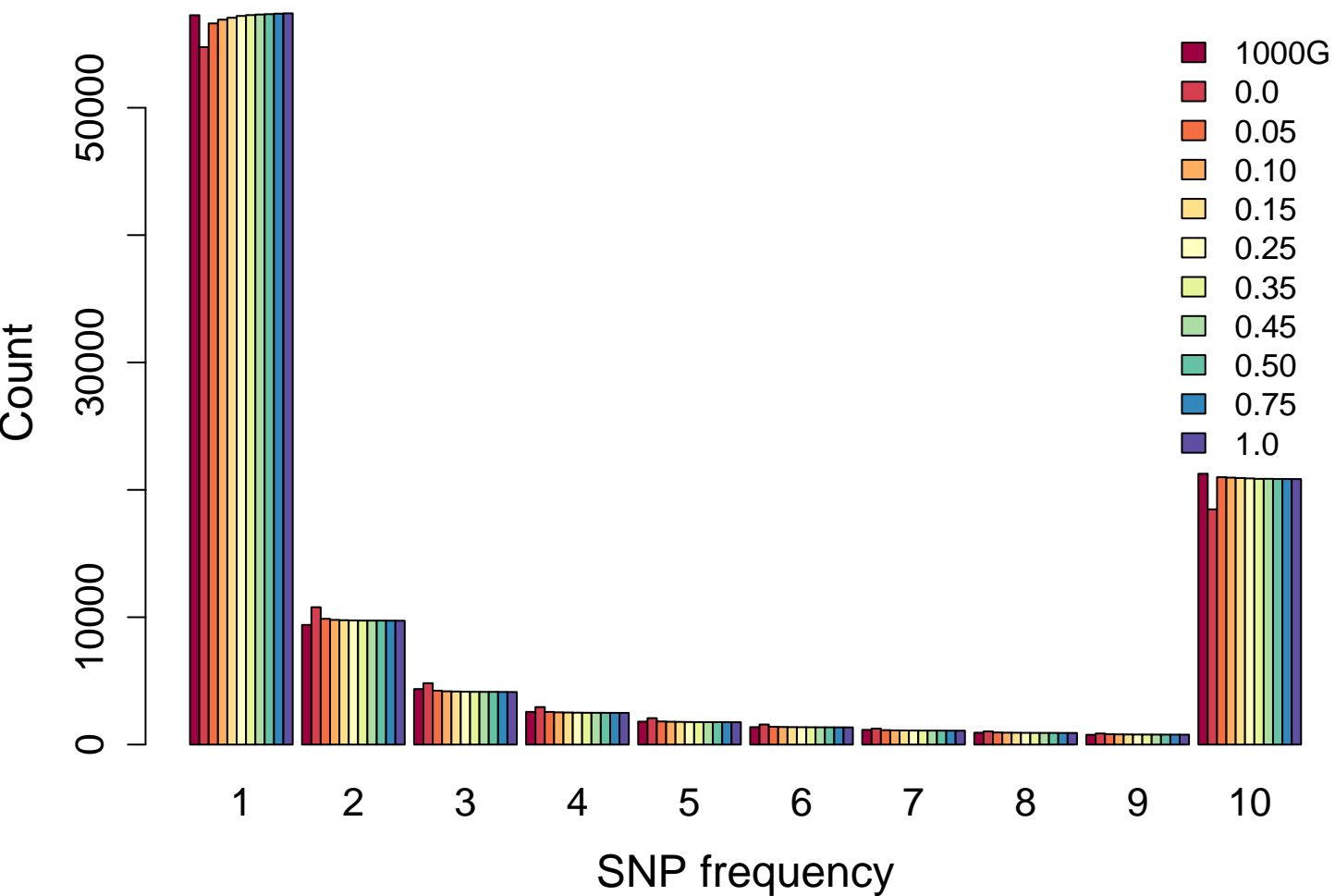

Supplement: S1 Fig — Results shown for each h value relative to the empirical SFS from the 1000G dataset. (PDF) [file pgen.1011198.s008.pdf]

## SFS for discrete model

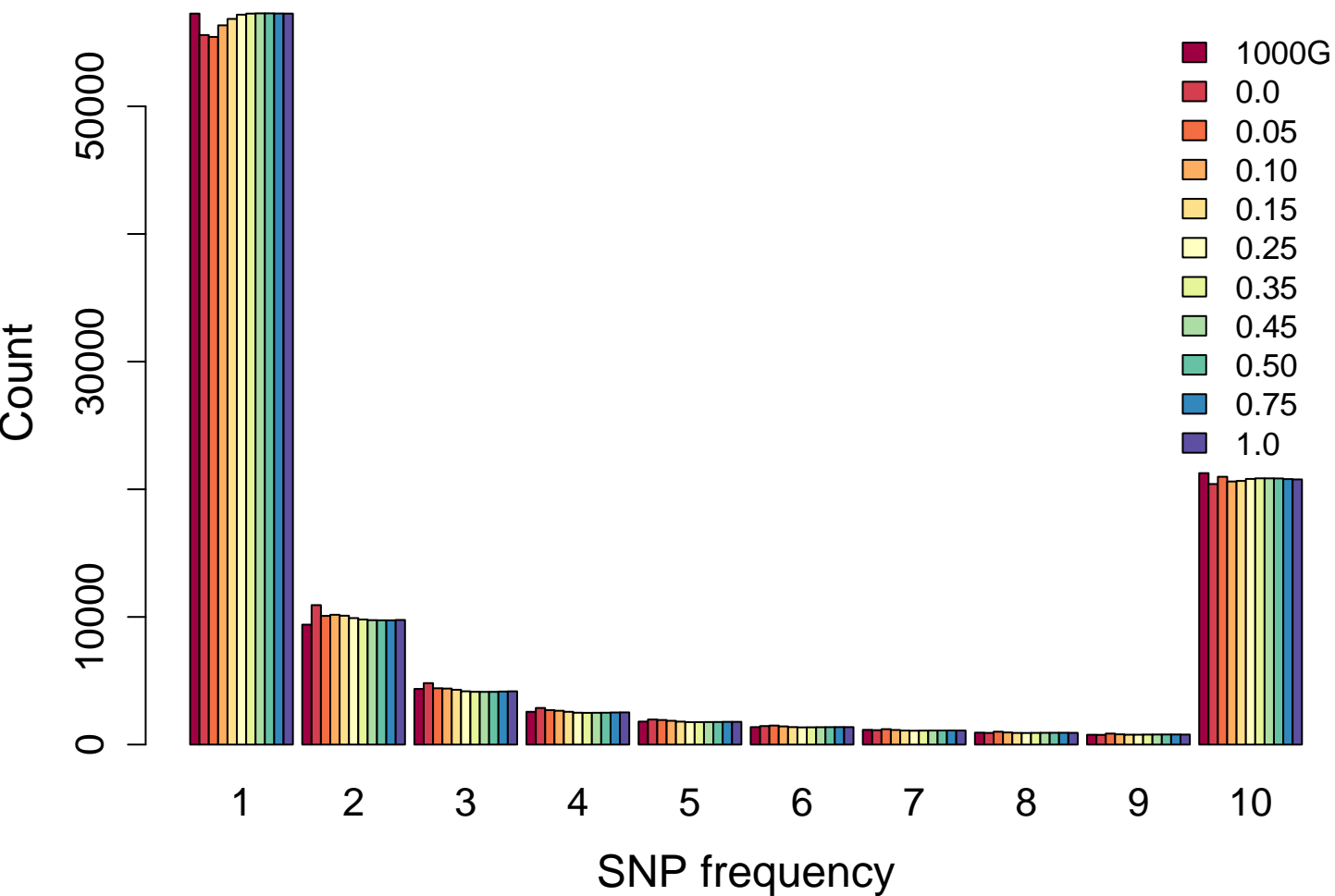

Supplement: S2 Fig — Results shown for each h value relative to the empirical SFS from the 1000G dataset. (PDF) [file pgen.1011198.s009.pdf]

**All models,  $n = 4096$** 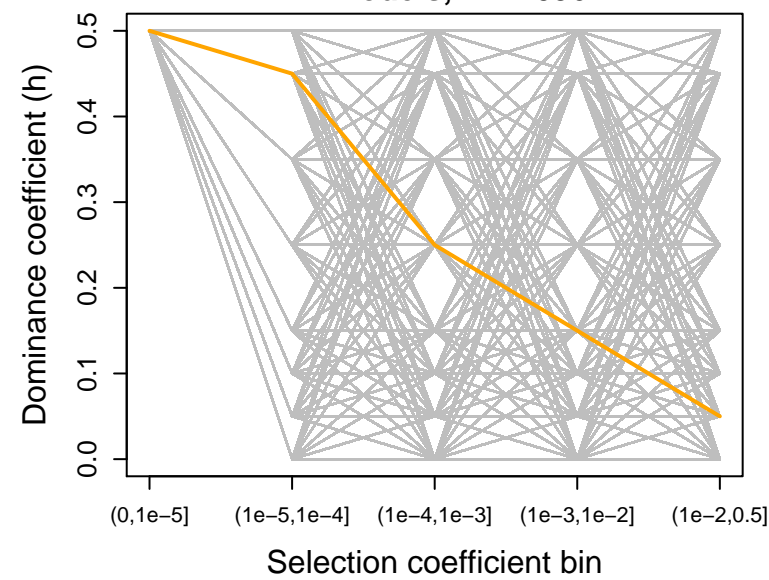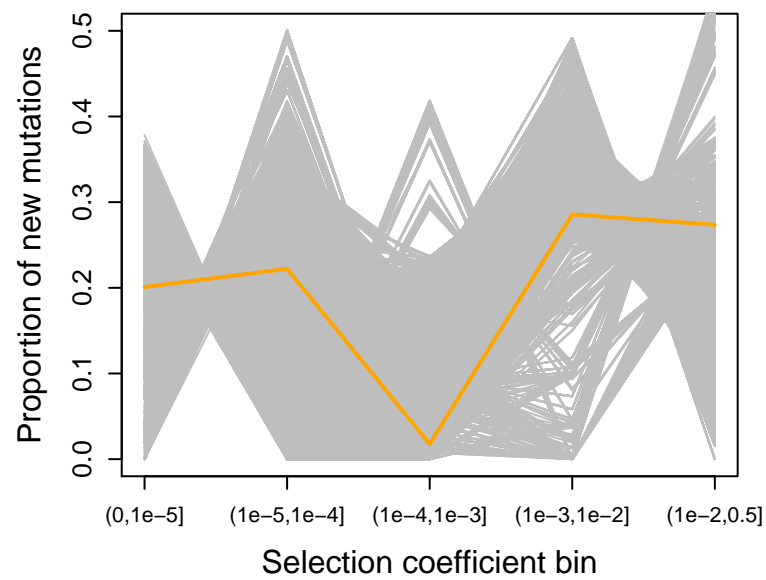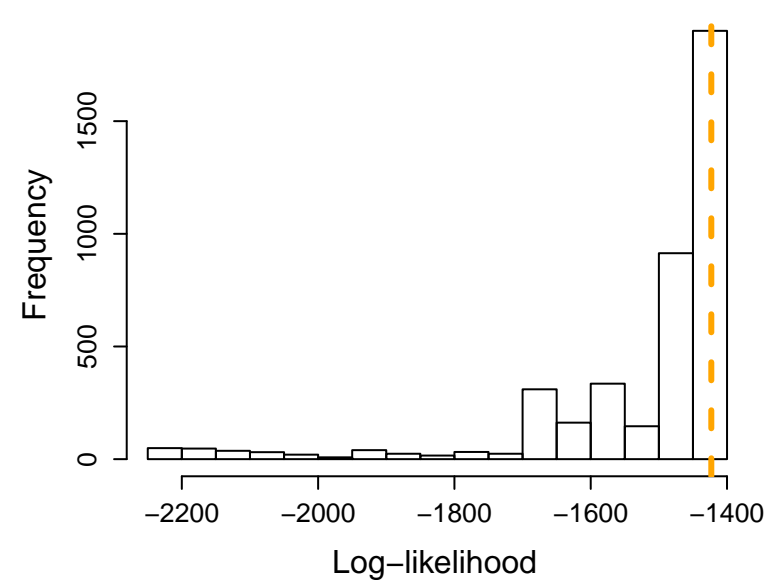**Models 4.74 LL away from MLE,  $n = 561$** 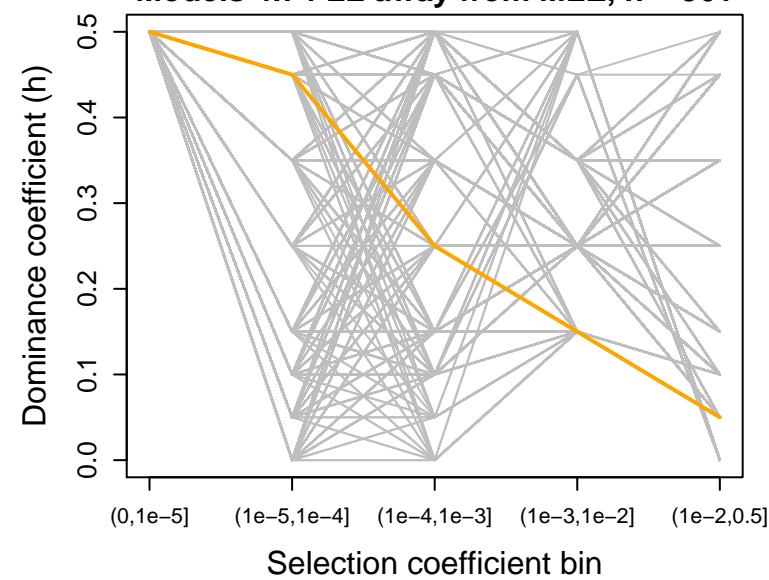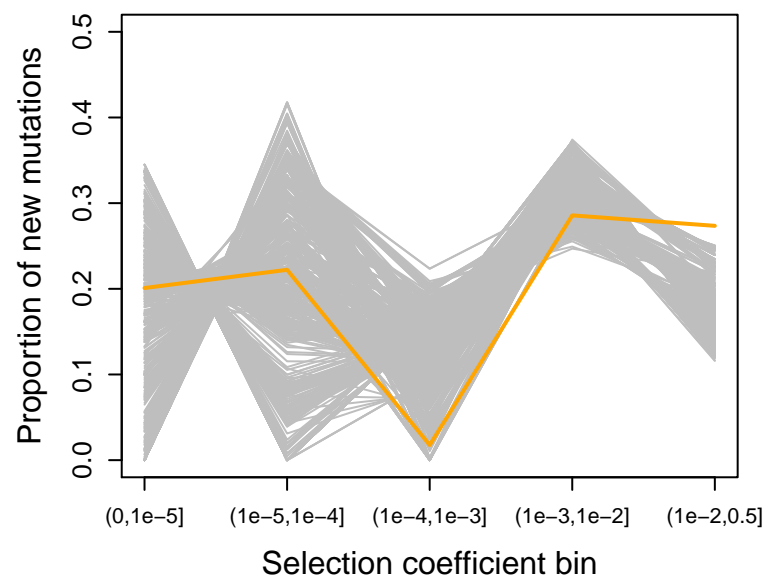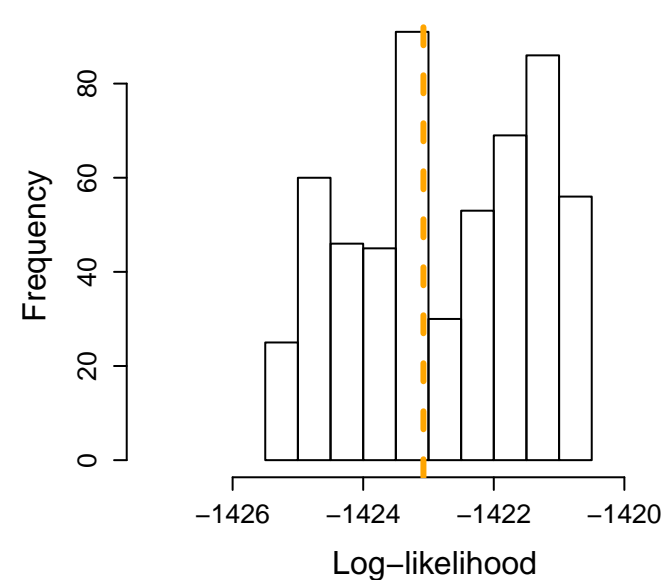**Models with monotonic decay of  $h$ ,  $n = 55$** 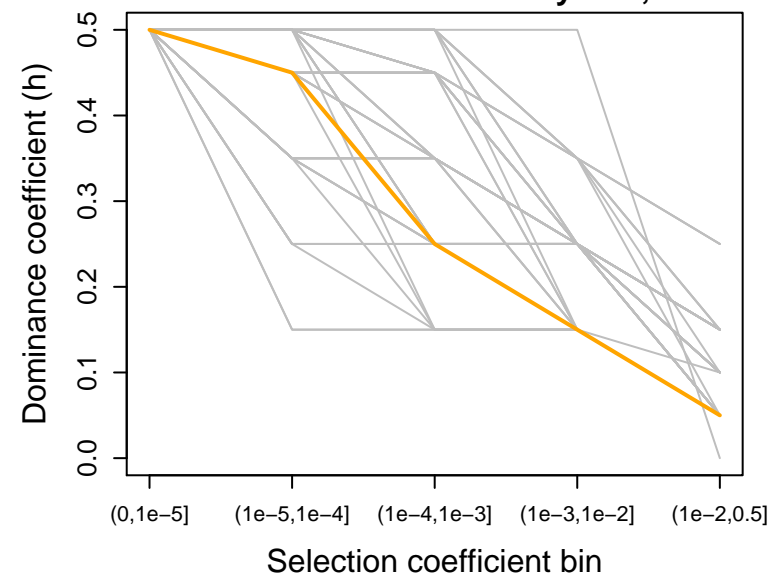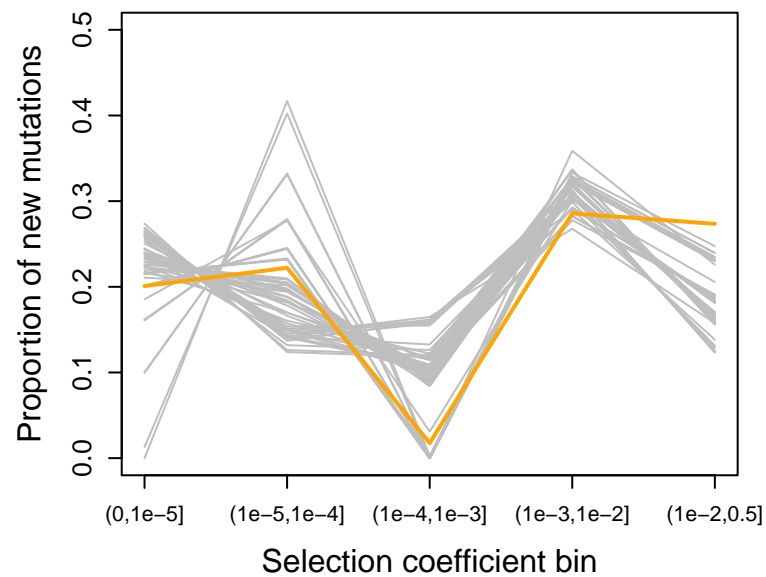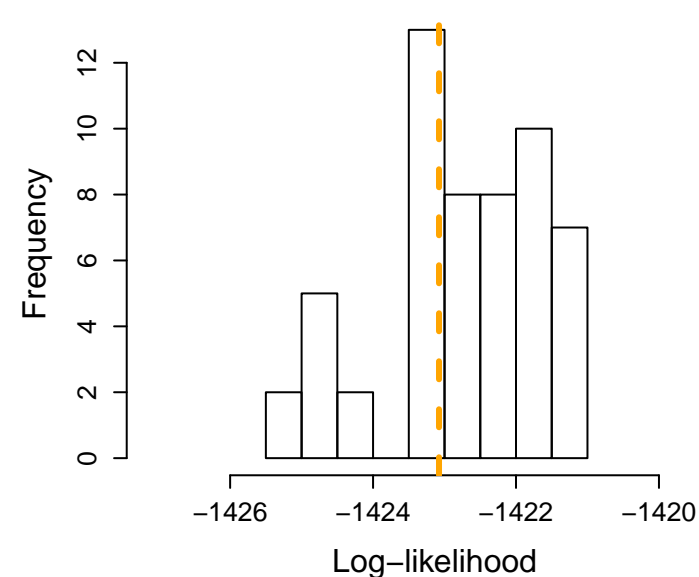

Supplement: S3 Fig — Dominance parameters (left column), selection parameters (middle column), and model fit (right column) for the true model are shown in orange. Note that the true model was 2.54 LL units away from the MLE and that the high LL models with monotonic decay (bottom row) have qualitatively similar dominance and selection parameters to the true model. (PDF) [file pgen.1011198.s010.pdf]

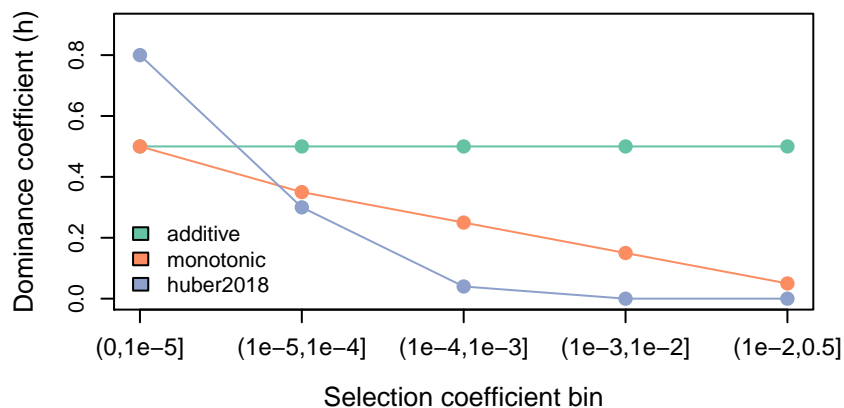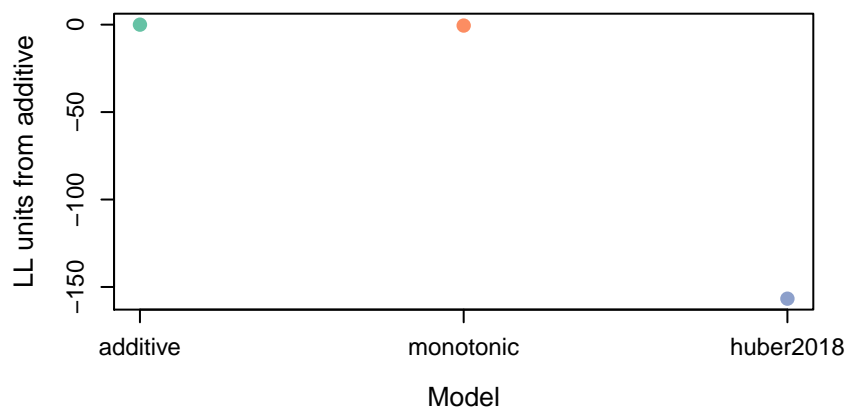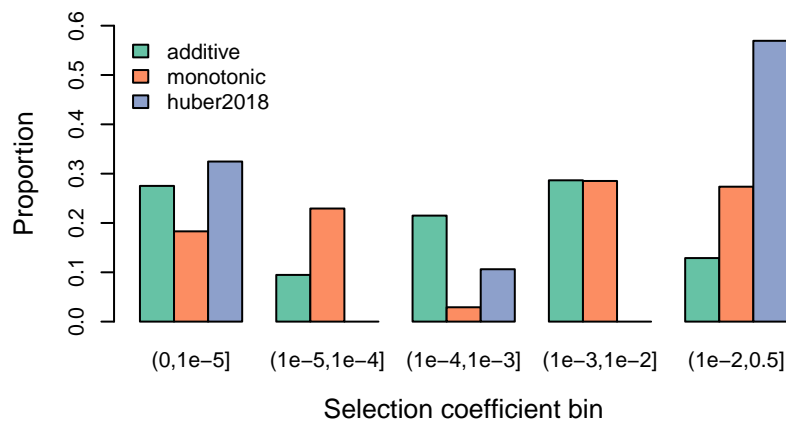

Supplement: S4 Fig — Top: Comparison of dominance parameters for models considered, including a fully additive model, a model with a monotonic decay in h that is within 1.92 LL units of the MLE, and the model from Huber et al. [25] estimated for Arabidopsis. Middle: Model fit in comparison to the additive model. Note that the additive model and monotonic models have similar log-likelihoods though the Huber et al. [25] model has a much worse log-likelihood. Bottom: Discrete DFE parameters estimated for each dominance model when fit to the human nonsynonymous SFS. Y-axis shows the proportion of mutations in each bin of the discrete DFE. (PDF) [file pgen.1011198.s011.pdf]

# nonsynonymous SFS

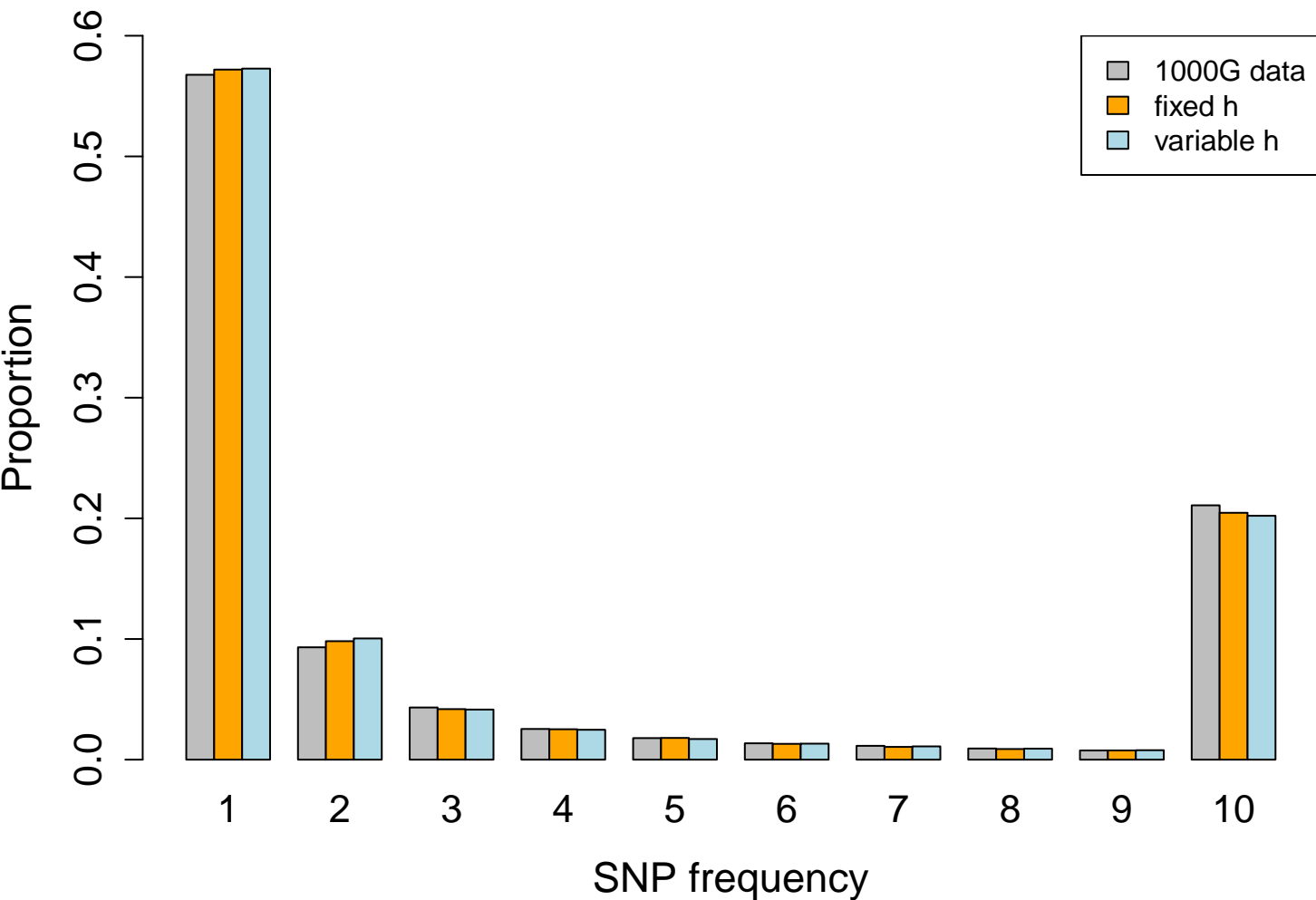

Supplement: S5 Fig — Both models assume a Strongly Recessive model (Table 2), however, in the ‘variable h’ model, h is allowed to vary for a given bin of s. Note that the SFS for fixed and variable h models are both quite similar and closely match the empirical 1000 Genomes Project data. (PDF) [file pgen.1011198.s012.pdf]

**A**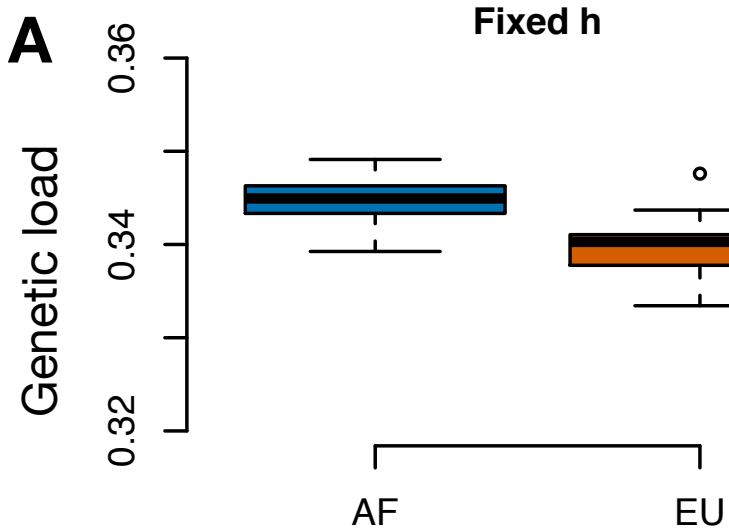**Variable h**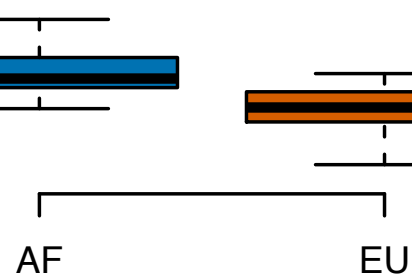**B**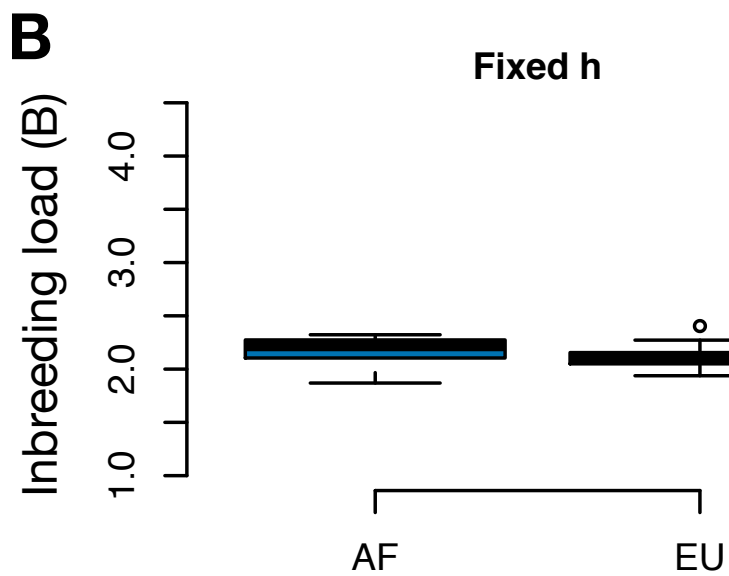**Variable h**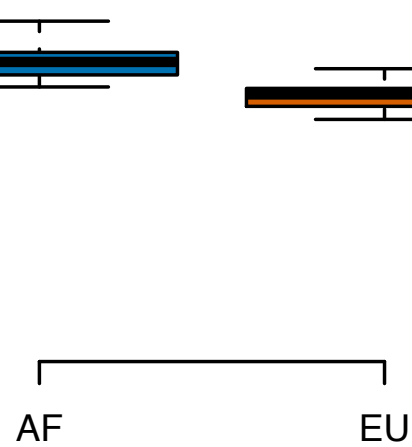**C**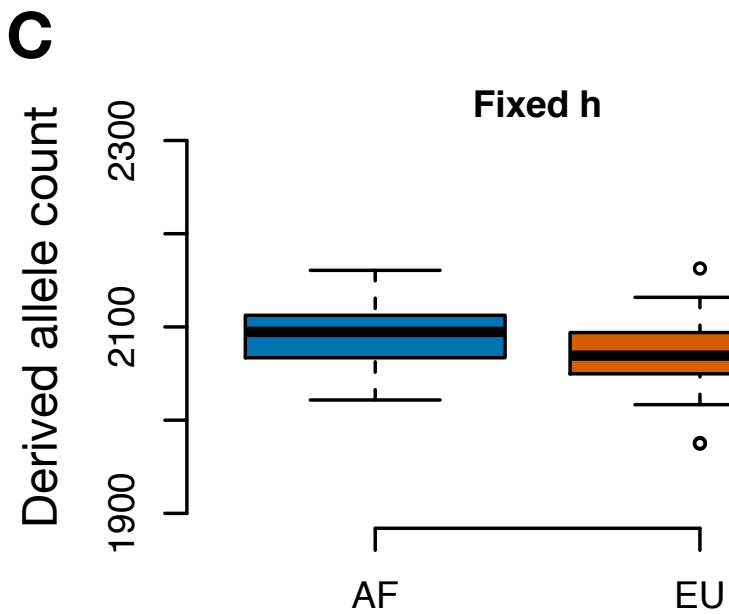**Variable h**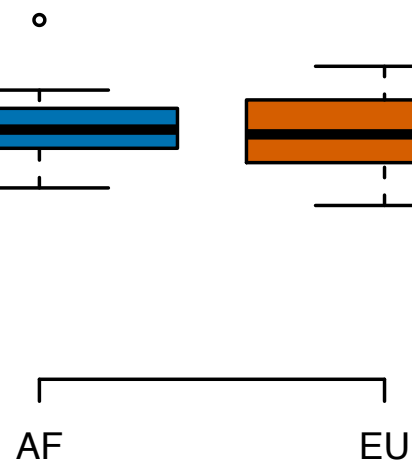

Supplement: S6 Fig — (A) Predicted genetic load in African and European populations. (B) Predicted inbreeding load in African and European populations. (C) Predicted derived deleterious allele count in African and European populations. Results are shown as boxplots summarizing output from 25 simulation replicates under each model. Note that the fixed h results on the left are from the Strongly Recessive model shown in Fig 4. (PDF) [file pgen.1011198.s013.pdf]

A

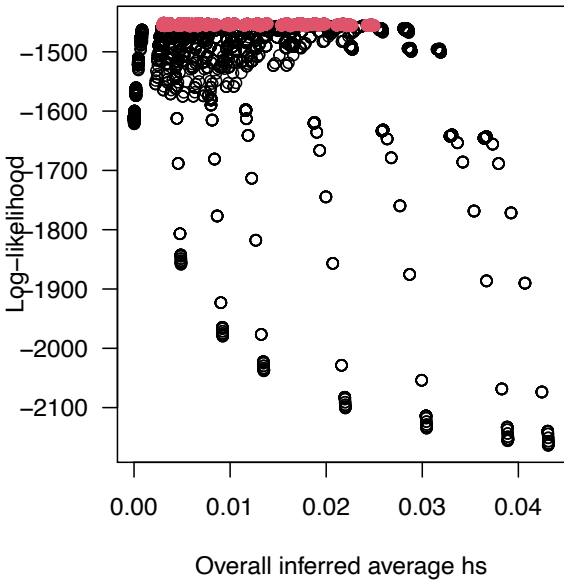

B

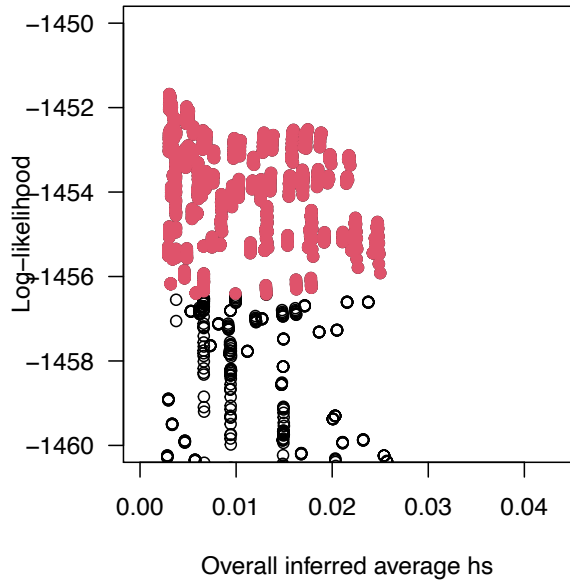

Supplement: S7 Fig — Inferred average h*s is calculated as the sum over all the bins of the DFE of the expected value of s for the bin multiplied by the value of h for that bin multiplied by the proportion of mutations inferred to be in that bin of the DFE. Each point represents a particular model. Red points denote those models with a log-likelihood <4.74 units below the fully additive model. These models fit the data well. (A) All models. (B) Zooming in to the models with the highest log-likelihood. (PDF) [file pgen.1011198.s014.pdf]

A

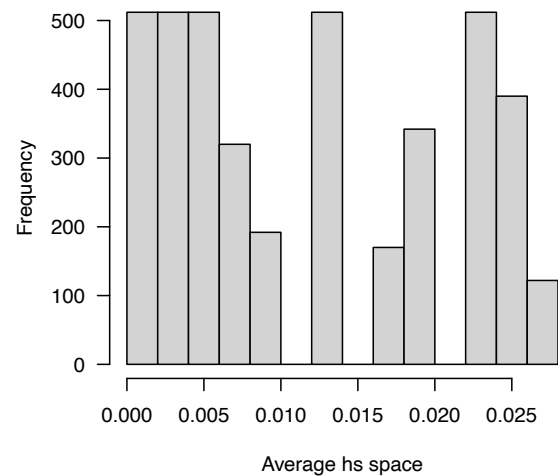

B

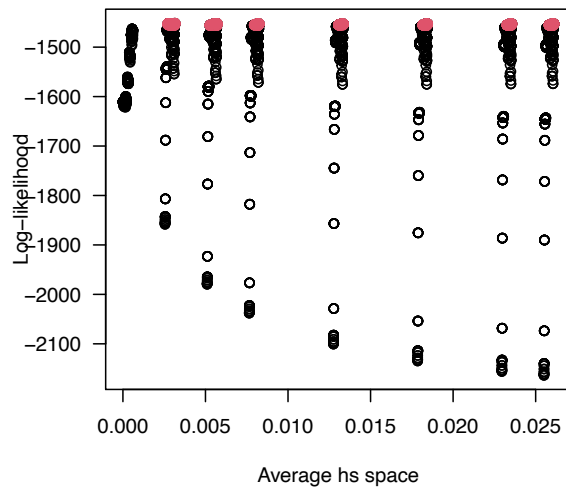

C

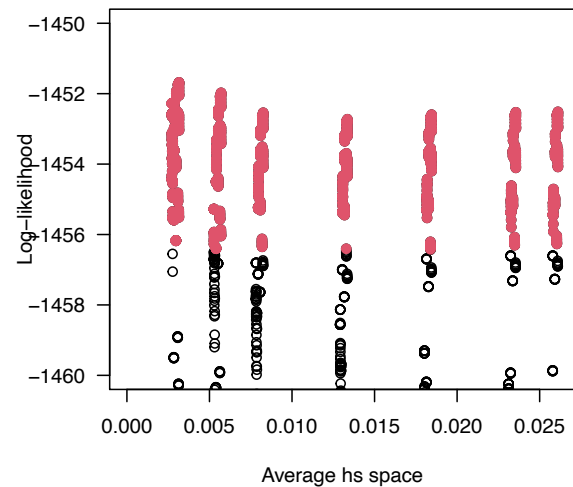

Supplement: S8 Fig — The average h*s space is calculated as the average over the 5 bins of the discrete DFE of the expected value of s for each bin multiplied by h for that bin. (A) Average h*s values of the 4096 models evaluated. Note that the space of average h*s is not exactly uniform. (B) However, models across the range of the average h*s space have high log-likelihood, indicating a good fit to the data. Red points denote those models with a log-likelihood <4.74 units below the fully additive model. (C) Same as (B), but zooming in on the top of the y-axis. (PDF) [file pgen.1011198.s015.pdf]
