# Supplementary material for: Constraining models of dominance for nonsynonymous mutations in the human genome
Source: PLoS Genet. 2024 Sep 20;20(9):e1011198. doi: 10.1371/journal.pgen.1011198 (PMC11446423; doi:10.1371/journal.pgen.1011198)
Supplement: Attachment — Submitted filename: response_to_reviewers_r1_submitted.pdf [file pgen.1011198.s016.pdf]

**Response to reviewers for Kyriazis and Lohmueller**

We are grateful to the editor and reviewers for providing us with useful feedback on our manuscript and inviting us to submit a revised version. Below, we detail our response to each reviewer comment in blue with line numbers that correspond to those in the unmarked (clean) copy of the manuscript.

Reviewer's Responses to Questions

**Comments to the Authors:**

**Please note here if the review is uploaded as an attachment.**

Reviewer #1: In their manuscript, “Constraining models of dominance for nonsynonymous mutations in the human genome”, Kyriazis and Lohmueller used a combination of modelling and simulations with publicly available human genome data to assess the fit of various dominance and selection models for nonsynonymous mutations in human populations as well as examine how dominance affects the relative genetic load burden of deleterious variation in ancestral African versus derived European human populations. The authors found that a wide range of h values could fit the data, a negative relationship between h s, and that relative genetic load burden in the non-African population was dependent upon the dominance model. The manuscript is clearly written and the topic itself is a timely and interesting contribution to the field. I have only a few comments that I think need addressing, which I have outlined in the major and minor points below:

**Response 1**

We are grateful for the positive feedback from the reviewer. Below, we detail changes made in response to the reviewer’s comments.

Major:

1. The authors used 432 individuals with European ancestry (described in lines 382–4), however, it is unclear where these individuals came from. Are they from the same or different populations? If it is the latter, did the authors check for potential population substructure (or is anything on this published), which might affect their results?

**Response 2**

We thank the reviewer for pointing out this important information on the ancestry and population structure in our sample. We have now added this information to the Methods on lines 450-460:

*This European sample includes individuals from five different European populations (Utah residents with Northern and Western European ancestry, British in England and Scotland, Finnish, Iberian populations in Spain, and Toscani in Italy), among which there is pronounced population structure [38] that could potentially impact DFE inference. However, previous simulation analysis by Kim et al. [27] demonstrated that such cryptic population structure does not impact the performance of Fitđaði, so long as the demographic model can fit the synonymous SFS. Additionally, this paper also showed that the DFE inferred using the European sample from the 1000 Genomes Project was similar to the DFEs inferred from a more homogenous sample from Denmark as well as from the NHLBIO GO Exome Sequencing Project (ESP) data, which consists of a heterogenous sample of individuals with European ancestry [27].*

Thus, although there is population structure within our sample, it should not impact our ability to infer the DFE given that our neutral demographic model yields a good fit to the synonymous SFS.

Minor:

1. Line 41 – At the beginning of the line, should "heterozygous" be "homozygous"?

**Response 3**

We thank the reviewer for catching this typo! It is now fixed.

2. Lines 134–153: There seem to be some typos in this sentence: "Given that we cannot reliably separate s and h for based on genetic variation data, we instead constrain the range of dominance models are consistent with the nonsynonymous SFS." It is hard to understand what the authors mean.

**Response 4**

We thank the reviewer for noting this issue! The corrected sentence is as follows:

*Given that we cannot reliably separate s and h based on genetic variation data, we instead constrain the range of dominance models that are consistent with the nonsynonymous SFS.*

Reviewer #2: Summary
It is generally accepted that dominance and selection coefficients are only weakly identifiable, such that a range of h values may be consistent with frequencies of deleterious alleles. It is not known how wide this range is and how it varies with s, and this is the knowledge hole the authors attempt to fill. This goal and the Poisson random field DFE inference methods used to achieve it are appropriate. However, the parameterization of the DFE makes interpretation difficult and may have serious statistical issues. In addition, the potential for dominance to vary for a given strength of selection is not considered or mentioned, making the authors conclusions less broad than would be desired.

**Response 5**

We thank the reviewer for this positive feedback. Below, we provide detailed response to these issues of DFE parameterization and variability in *h* for each bin of *s*. We believe that these revisions have helped to broaden our conclusions and validate our analysis approach.

Major comments
Analyses of non-synonymous variation in humans typically assume additivity of selection coefficients (h=0.5) between heterozygous and homozygous genotypes. Simultaneously, it is appreciated that the identifiability of moderate variation in h is limited from allele frequency data. The authors note that this implies there is a range of h and s combinations that should be compatible with the distribution of allele frequencies. The aim of this manuscript is to determine where the boundaries of that compatible h-s space are and to provide a sense for the consequences of such a plausible departure from additivity. To this end the authors fit a large set of models to the distribution of missense variant frequencies from nearly all protein coding genes in European ancestry individuals in 1000 Genomes. Models with similar likelihoods to the best fit are considered to be plausible models of dominance. Using this approach, they report that a global h as low as 0.15 is consistent with the data and that h can be as low as 0.05 for strongly selected alleles.

I agree with both the general need for such a study and the general approach taken by the authors. Having a sense of the relevant parameter space would be a great resource for researchers looking to relax additivity assumptions. However, I find two serious issues with the manuscript in its current state. The first is that parameterizing and presenting models in terms of h and s (the selection coefficient against homozygotes) leads to conceptual and statistical problems. As the authors note, the shape of the SFS is largely determined by h*s, the heterozygote selection coefficient. The distribution on h*s is the foremost object being fit by DFE inference, and is what most people think of as the DFE (due to h=0.5 assumption). The authors treat the distribution on s as the DFE. This can be confusing, and it also makes any plot of DFE changes difficult to interpret since it isn't clear what differences are due to dominance versus an attempt to keep the h*s distribution relatively constant.

**Response 6**

We thank the reviewer for raising these important issues. While much of what determines the shape of the SFS is *h*s*, there are both practical and philosophical reasons why considering *h* and *s* as separate parameters in this context is preferable.

In terms of our philosophical justifications, our view is that the DFE should be thought of as the distribution of *s*, and that dominance parameters can be considered a separate quantity to be estimated. Considering *s* and *h* as separate parameters has a long history in the literature (e.g., Simmons & Crow 1977; Deng & Lynch 1996; Williamson et al. 2004; Agrawal & Whitlock 2011; Huber et al. 2018), given that they ultimately reflect two distinct properties of mutations. Although we agree that many recent papers have focused on estimating the compound parameter *h*s* (e.g., Cassa et al. 2017; Agarwal et al. 2021, 2023), we believe this is more a consequence of acknowledging the difficulty associated with separately estimating *s* and *h*, rather than a fundamental belief that *h*s* is ultimately the parameter of interest (e.g., see Fuller et al. 2019 for a discussion of this).

On a more practical level, a major motivation for our paper is to provide the broader population genetics community with a reasonable set of *s* and *h* parameters to be used for modelling studies. As the reviewer is undoubtedly aware, a model *s*=0.1 and *h*=0.01 for deleterious mutations will have vastly different behavior in terms of the dynamics of genetic load and inbreeding depression compared to a model with *s*=0.002 and *h*=0.5, even though in both cases *h*s*=0.01. Thus, although the ability to disentangle *s* and *h* remains limited in our analysis, we still believe there is practical value in providing a range of reasonable parameter estimates to the broader community. For instance, in the conservation genetics community, there has been an ongoing debate on selection and dominance parameters for use in population viability modelling (see Kyriazis et al. 2023 *Am. Nat.* for discussion), and we believe that the results of our present analysis can provide some clarity to this topic.

That said, the reviewer's comment inspired us to more fully investigate the values of *h*s* in the discrete DFEs and how average *h*s* varies for models that fit the data. Figure S7 (pasted below) of the revised manuscript shows that discrete DFEs with average *h*s* values ranging from 0.003 to 0.025 fit the observed SFS well. Importantly, models that have very small average values of *h*s* do not fit the data, consistent with our other analyses suggesting that models where many deleterious mutations are highly recessive do not fit the data. It is also noteworthy that there are many models having an average *h*s* within the range of 0.003 to 0.025 also do not fit the data well. The reason for this is that different parameter values from the joint distribution of *h* and *s* can lead to similar average *h*s* values. As such, average *h*s* does not fully capture the joint distribution of *s* and *h*, making it appropriate to consider the DFE as we do here, rather than exclusively focusing on *h*s*.

We added the following discussion of these issues in our revised manuscript on lines 429-436:

*Ultimately, any approach for inferring dominance and selection parameters from allele frequency data will have limited power to disentangle h*s in outbreeding species [24,25]. Indeed, we find that models with a good fit to the data have an average h*s in the range of 0.003-0.025 (**Fig. S7**). However, not all DFE and dominance models with an inferred average h*s in this range fit the data well, as there are many combinations of s and h values for different bins of the DFE that yield similar average h*s values. Furthermore, our aim of separately considering h and s parameters in this study has practical benefits in that it can yield parameter combinations that can be used in simulation studies (**Fig. 4**).*

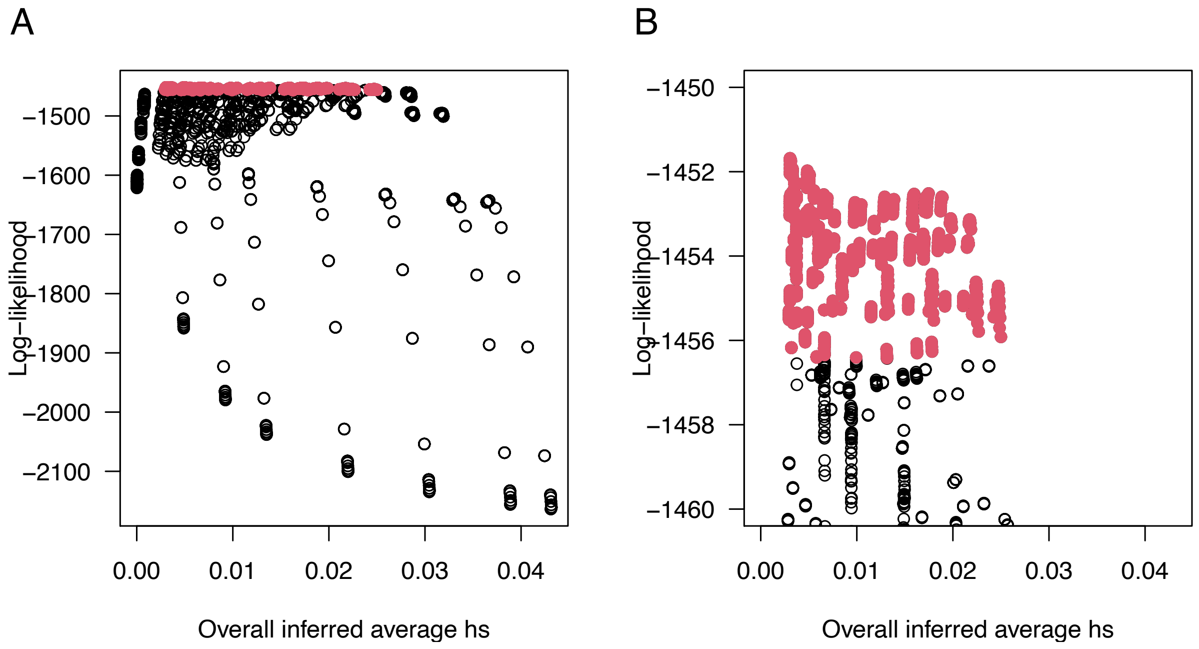


**Figure S7: Inferred average *h*s* for each of the 4096 discrete DFE/*h* models considered.** Inferred average *h*s* is calculated as the sum over all the bins of the DFE of the expected value of *s* for the bin multiplied by the value of *h* for that bin multiplied by the proportion of mutations inferred to be in that bin of the DFE. Each point represents a particular model. Red points denote those models with a log-likelihood <4.72 units below the fully additive model. These models fit the data well. (**A)** All models. **(B)** Zooming in to the models with the highest log-likelihood.

A potentially larger statistical problem with the (h,s) parameterization arises in the context of discrete DFE models. These models assume a uniform distribution on s within approximately each factor-of-ten range from s=1e-5 upwards. The relative weights of these bins are fit, along with a corresponding h, during the inference procedure. Allowing h to vary for each bin means that the model space of h*s distributions changes as well. For instance, holding all other bins at h=0.5, decreasing h for the first bin will create a gap between the first and second bins in h*s space. In general, changing h values creates gaps and overlaps which affect what h*s distributions are possible. It is therefore not clear whether likelihood differences between

models result from aspects of the SFS informative about dominance, or whether they are due to changes in the h*s model space. Since all the main results rely on discrete DFE models, it isn't clear whether the conclusions of the manuscript are robust. Repeating the analysis by putting the bins on h*s instead of s would likely solve this problem, though other robustness checks are possible.

**Response 7**

We thank the reviewer for asking us to investigate this issue. As outlined in **Response 6** above, we believe that considering *s* and *h* as separate parameters in our analysis is appropriate for the aims of our paper. Here, we further examine the *h*s* model space considered by the discrete DFE to determine whether there may be artefacts in our inference approach, as suggested by the reviewer.

We computed the "average *h*s*" of each of the 4096 discrete models being considered in our inference. To do this, we multiplied the value of *h* for a particular bin by the expected value of s for that bin. Then we averaged this quantity across the 5 bins of the discrete DFE. This is what we believe the reviewer is referring to as the "*h*s* model space." Figure S8A (pasted below) shows a histogram of these average *h*s* values considered. Note that the space of models considered includes average *h*s* values ranging from $5 \times 10^{-7}$ to 0.0261. As the reviewer suggested, the space of average *h*s* values considered was not exactly uniform. There are some gaps in the distribution (e.g. around 0.01). However, this is not a problem for our inference, as it considers points above and below these gaps. Figure S8B-C shows the log-likelihoods for different average *h*s* values in the model space. There are many average *h*s* values with high log-likelihoods that match the data well. Only values <0.0027 can be exclude as having a poor fit to the data. It is not clear to us what having an additional point in the *h*s* space of say, 0.01-0.012 would add to this analysis.

We discuss some of these results in the Methods section of the revised manuscript (on lines 580-591):

*One potential concern with parameterizing a discrete DFE in terms of a uniform distribution of s within each bin and a specific value of h for that bin is that different values of h will result in models having different h*s values. As h*s largely influences the SFS, the fit of different models could potentially be driven by changes in h*s model space, rather than dominance, per se. To check this, we determined the location of each of the 4096 models in average h*s model space. To do this, we multiplied the value of h for a particular bin of the discrete DFE by the expected value of s for that bin. Then we averaged this quantity across the 5 bins of the discrete DFE. There are gaps in the average h*s model space that we considered (**Fig. S8A**). However, this does not appear to influence our results as models with an average h*s in the range of $5 \times 10^{-7}$ - 0.026 all have high log-likelihoods, indicating a satisfactory fit to the data (**Fig. S8B,C**). Further, not all models within the average h*s space $5 \times 10^{-7}$ - 0.026 fit the data well, reflecting the fact that models with different combinations of h and s can have similar average values in h*s space.*

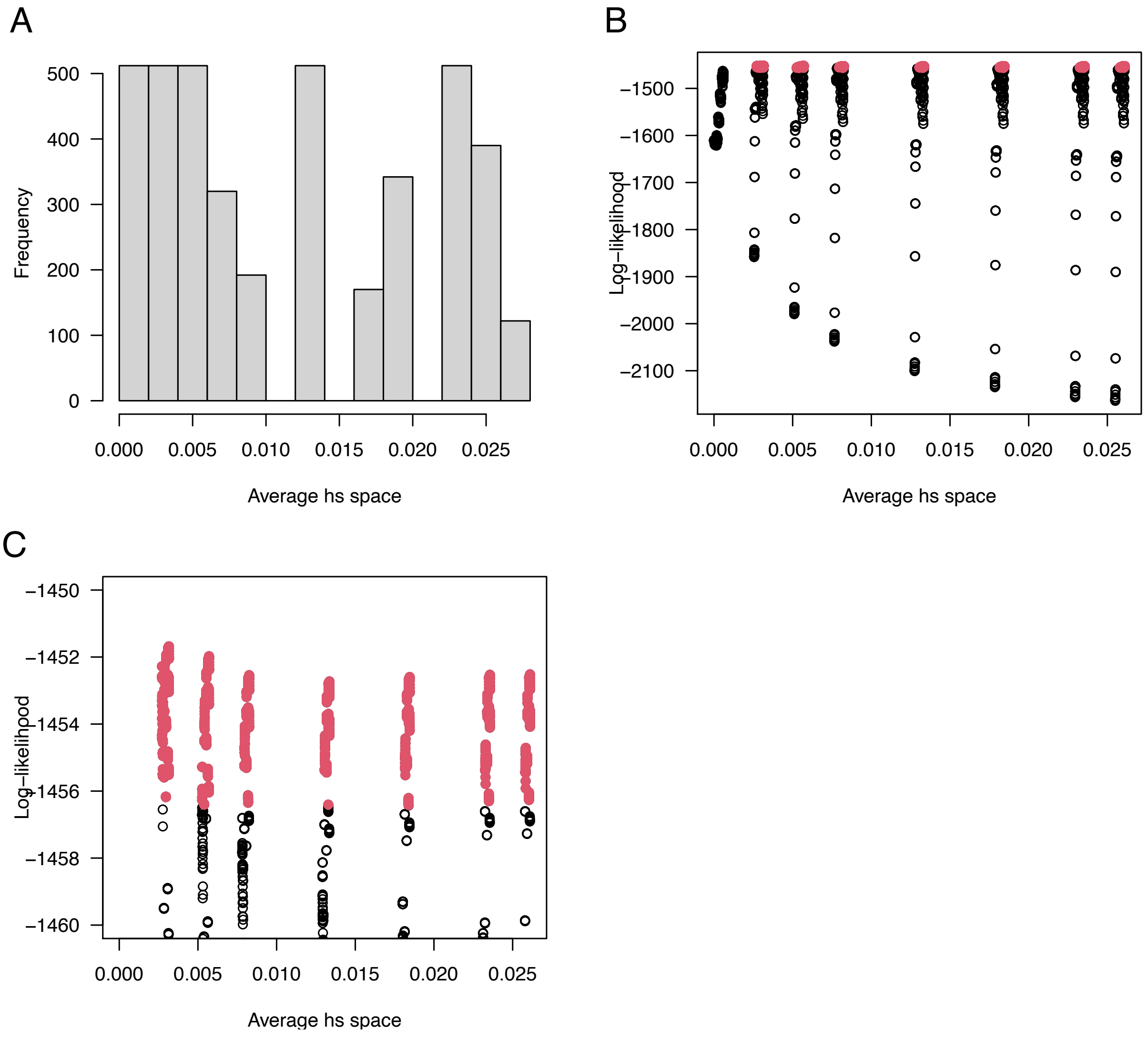


**Figure S8: Examination of the average *h*s* model space.** The average *hs* space is calculated as the average over the 5 bins of the discrete DFE of the expected value of *s* for each bin multiplied by *h* for that bin. **(A)** Average *h*s* values of the 4096 models evaluated. Note that the space of average *h*s* is not exactly uniform. **(B)** However, models across the range of the average *h*s* space have high log-likelihood, indicating a good fit to the data. Red points denote those models with a log-likelihood <4.74 units below the fully additive model. **(C)** Same as **(B)**, but zooming in on the top of the y-axis.

Finally, as another robustness check, we now validate our inference procedure on simulated data where the true dominance and selection parameters are known. This analysis is described in the Methods on lines 572-579:

*Given the large parameter space of models being fit, we assessed using simulated data whether this inference approach would be able to identify the true model from the large set of 4096 models being fit. To do this, we simulated nonsynonymous sites across 22 autosomes (totaling*

*~30Mb of sequence) under the single population demographic model from Kim et al. [26] using SLiM v4.0.1 [39–41]. We assumed selection and dominance parameters from the Strongly Recessive model (Table 2) and all other parameters were the same as our main simulation analysis described below. We then used the resulting nonsynonymous SFS as input for the inference approach described above.*

And in the Results on lines 212-216:

*Given the challenges of identifying the 'true' model in this large parameter space, we validated our approach on simulated data, finding that the true model was within 2.54 LL units of the MLE (see* ***Methods; Fig. S4****). Based on this finding, we used a cut-off of 4.74 LL units (the asymptotic 95% confidence interval for a model with 4 free parameters) to designate models with good fit.*

We also include the new Figure S4 with these results below:

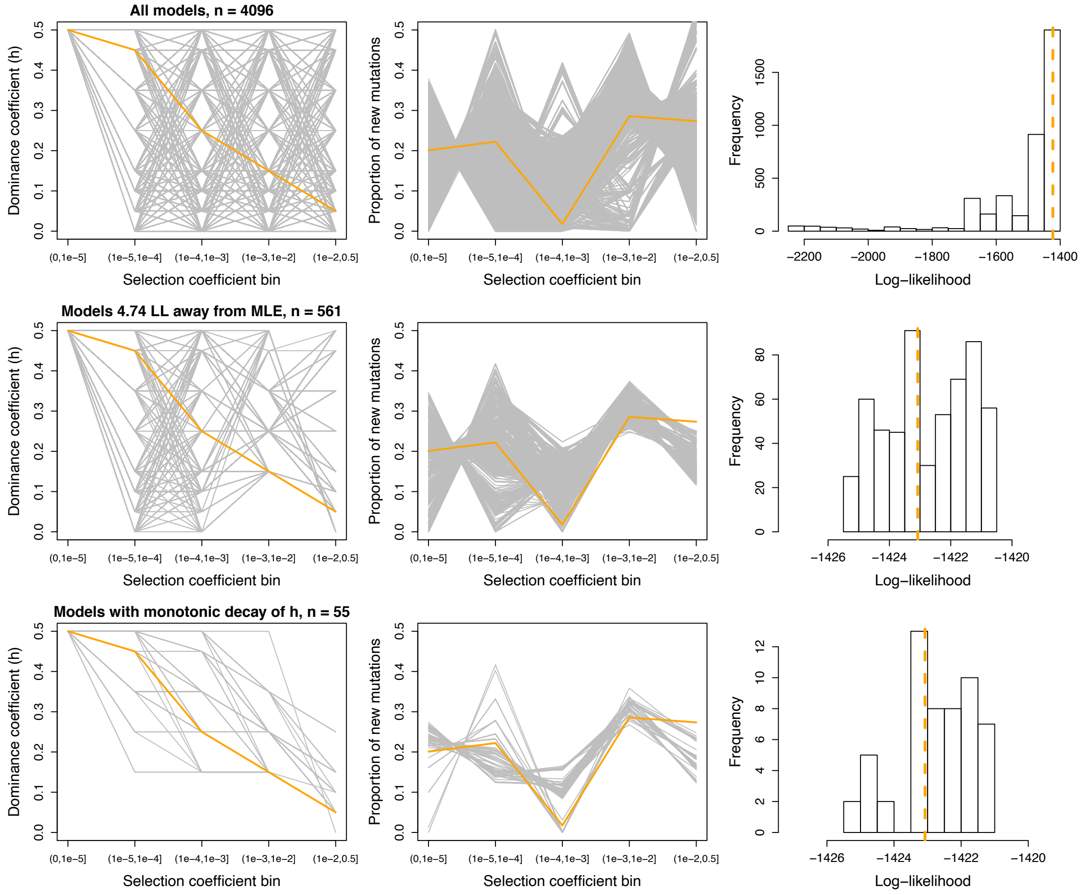


**Figure S4: Performance of *h* and *s* inference approach on simulated data.** Dominance parameters (left column), selection parameters (middle column), and model fit (right column) for the true model are shown in orange. Note that the true model was 2.54 LL units away from the MLE and that the high LL models with monotonic decay (bottom row) have qualitatively similar dominance and selection parameters to the true model.

The second major issue is the lack of consideration for or mention of the possibility that dominance may differ among mutations with the same s. This seems to be exactly the scenario suggested by human Mendelian genetics. If we consider genes causing Mendelian disorders to be in the same bin of fitness effects, both h=0 and h=1 examples clearly exist. Identifying loci, genes, and gene sets with dominance effects on traits or fitness has been a major focus of previous work in this field (see Palmer et al. 2023 Science and Balick et al. 2022 AJHG for two recent examples). Huber et al. 2018 Nat Comm did suggest that deterministic h-s models capture the average relationship when h is random, but isn't clear that the average h|s is what one should care about for the purposes of the manuscript. If so, this should be justified. Is it the proportion of strongly recessive mutations or the average h that determines inbreeding load, for instance. What information about h|s is identifiable or possible to bound? The authors should address whether their conclusions are robust to variance in h.

**Response 8**

We thank the reviewer for noting the important issue of a variable *h* for a given *s*, which we did not consider in our original draft. Indeed, we agree that understanding the impact of varying *h*|*s* is an important and interesting question in the context of our results. To better explore this topic, we conducted simulations where we evaluate the impact of variable *h* on both the SFS as well as the predicted genetic load and inbreeding load. These results are detailed in the Results on lines 323-348:

***The impact of variable h for a given s***

*All of the dominance models considered so far assume a single h for a given s. However, it is well appreciated from studies of Mendelian disorders that loci with similar phenotypic effects can range from dominant to fully recessive [47]. Moreover, experimental studies in organisms such as yeast and Drosophila also support the possibility that h may vary for a given s [12,48]. To explore the impact of variable h in the context of our results, we ran simulations under the Strongly Recessive model from above; however, we allowed h for each bin of s to be drawn from a uniform distribution bounded by 0 and 2*$\bar{h}$. For instance, in the case of the strongly deleterious bin where $\bar{h}$=0.05, h could range from 0 to 0.1.*

*With this model, we first examined the impact of variable h on the predicted nonsynonymous SFS (**Fig. S5**). We observed no noticeable differences between the predicted nonsynonymous SFS when comparing models with fixed and varying h, and both models closely matched the observed SFS from the 1000G data (**Fig. S5**). Thus, this result demonstrates that modest variability in h for a given s might not impact overall patterns of genetic variation, implying that our inference approach can infer the average h for each bin of s even when variability is present.*

*We next employed this Strongly Recessive model with variable h to explore the potential impact of variable h on genetic load and inbreeding load. Here, we find that our previous qualitative finding for the Strongly Recessive model (**Fig. 4**) of diminished genetic load and inbreeding load in non-African populations was recapitulated under a model with variable h (**Fig. S6**). However, the magnitude of genetic load was shifted slightly down, whereas the*

*magnitude of the inbreeding load was greatly shifted up (mean B=~3.5 across populations;* ***Fig. S6****). This may be due to the increased extent of mutations that have the potential to be highly recessive (h<0.05) in this model, given that all deleterious mutation classes were assumed to have a lower bound of h=0.0. Overall, these results demonstrate that variability in h may have important impacts on genetic load and inbreeding depression, suggesting that future studies should aim to better parameterize variability in h for deleterious mutations in humans and other species.*

In addition, the Supplemental Figures with the relevant results are pasted below:

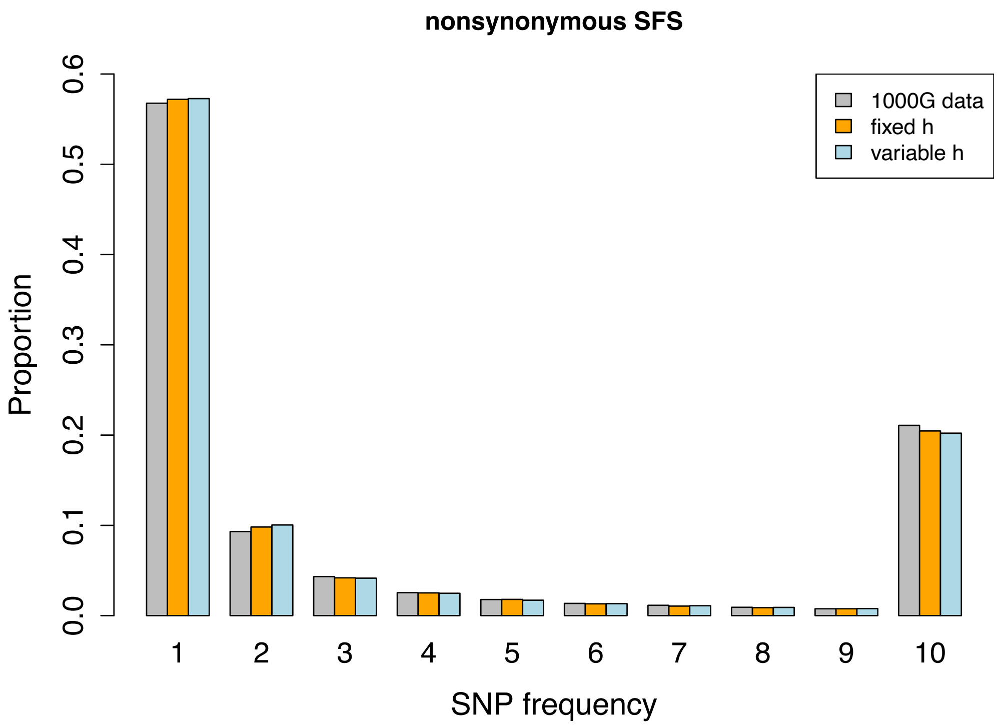


**Figure S5: Comparison of the SFS between models where *h* is fixed for a given *s* to models where h can vary.** Both models assume a Strongly Recessive model (Table 2), however, in the 'variable *h*' model, *h* is allowed to vary for a given bin of *s*. Note that the SFS for fixed and variable *h* models are both quite similar and closely match the empirical 1000G data.

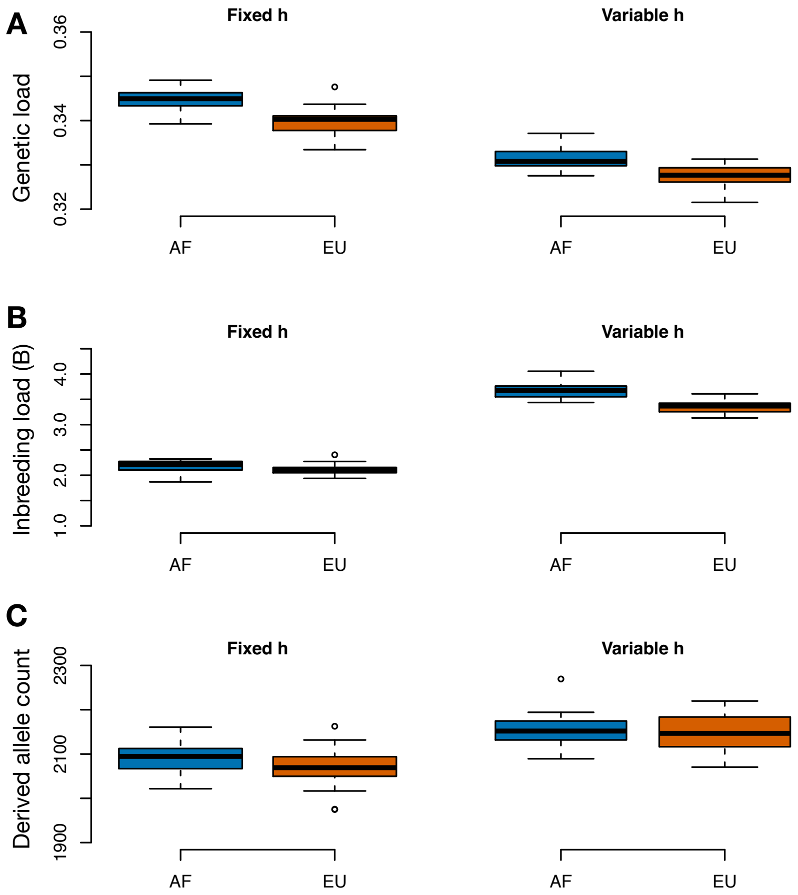


**Figure S6: Simulation results comparing predicted genetic load, inbreeding load, and derived allele count for African (AF) and European (EU) populations under a model where *h* can vary for a given *s*.** (A) Predicted genetic load in African and European populations. (B) Predicted inbreeding load in African and European populations. (C) Predicted derived deleterious allele count in African and European populations. Results are shown as boxplots summarizing output from 25 simulation replicates under each model. Note that the fixed *h* results on the left are from the Strongly Recessive model shown in Fig. 4.

Minor issues
When defining h in the introduction, a definition of s as specifically the homozygous selection coefficient should also be given.

**Response 9**

We thank the reviewer for noting this issue. We intentionally left the definition of *s* vague in the Introduction, given that Fitdadi in fact assumes the homozygote fitness to be 1-2*s* whereas SLiM assumes 1-*s*. Instead, we now detail this parameterization in the Results, separately for the Fitdadi results (on lines 163-164) and SLiM results (on lines 292-294).

Balick et al. 2022 AJHG should also be in the introduction as a study that used similar SFS

methods without the demography or selfing tricks, and reported evidence for strong recessive selection on some gene sets.

**Response 10**

We thank the reviewer for this recommendation. We added the following text to our Introduction to include some discussion of this study:

*In a follow up study, these authors also attempted to identify genes under recessive selection in the human genome from allele frequency data [11]. Their analysis demonstrated that, although there is little power to identify individual genes under recessive selection, such genes can be detected in aggregate. Thus, although some recent progress has been made in furthering our understanding of dominance in humans, numerous fundamental aspects remain poorly understood.*

84: It would be nice to expand briefly on what previously used ad hoc dominance models have been used given that this is specifically what the authors propose to improve upon.

**Response 11**

We thank the reviewer for this idea, as we agree these ad hoc dominance models are an important point of comparison for our results. We added the following text to the Introduction (lines 133-145) with detailed discussion of these models:

*In the relative absence of information about dominance in humans or vertebrates, many studies instead opt to use ad hoc dominance parameters for modelling deleterious variation (e.g., [2,6]), or explore only the extreme cases of additive and fully recessive mutations (e.g., [9,30]). For instance, Henn et al. [6] formulated an h-s relationship for modelling genetic load in humans that was highly recessive, where h declines below 0.05 when |s| increases beyond ~1e-3. This h-s relationship parameterization has since been employed by a number of other studies [2,31–33], though a recent analysis showed that such a highly recessive model is not consistent with measures of inbreeding depression in humans [34]. By contrast, other recent studies have employed much less recessive dominance parameters for modelling genetic load in wild mammals [35,36], where h declines below 0.05 only as |s| increases beyond ~0.2. However, these parameters have also shown to be inconsistent with available evidence in humans and other mammals [34]. Thus, a very basic practical need for parameterizing evolutionary simulations is a set of realistic selection and dominance parameters that are directly inferred from genetic variation datasets.*

146: I think it is fine, but the use of 1000 Genomes European data should be justified in a world where largely and more ancestrally homogeneous samples are available.

**Response 12**

We thank the reviewer commenting on this issue, which was also noted by other reviewers. Although there is undoubtedly population structure in this European sample from the 1000 Genomes Project, previous analysis in Kim et al. 2017 demonstrated that such population structure does not impact inference of the DFE so long as the demographic model yields a good fit to the synonymous SFS. We now comment on this issue in more detail in the Methods (on lines 450-460):

*This European sample includes individuals from five different European populations (Utah residents with Northern and Western European ancestry, British in England and Scotland, Finnish, Iberian populations in Spain, and Toscani in Italy), among which there is pronounced population structure [38] that could potentially impact DFE inference. However, previous simulation analysis by Kim et al. [27] demonstrated that such cryptic population structure does not impact the performance of Fitðaði, so long as the demographic model can fit the synonymous SFS. Additionally, this paper also showed that the DFE inferred using the European sample from the 1000 Genomes Project was similar to the DFEs inferred from a more homogenous sample from Denmark as well as from the NHLBIO GO Exome Sequencing Project (ESP) data, which consists of a heterogenous sample of individuals with European ancestry [27].*

165: When describing the discrete DFE in the main text, it should be stated that s is uniform with each bin.

**Response 13**

We thank the reviewer for this recommendation. We have added this information on lines 186-188 in the Results:

*The discrete DFE quantifies the proportions of mutations in each bin, where s is uniformly distributed within each bin.*

Reporting model fits using log likelihoods in the main text is somewhat difficult to read given that each instance contain 6 digits. The authors should consider ways to simplify this such as distance from a best-fitting additive model.

**Response 14**

We thank the reviewer for this suggestion on the presentation. For our presentation of the gamma models, we now report the distance from the best-fitting additive model. Although we considered making the same change for the discrete DFE results, reporting the difference in log-likelihood from the additive model made interpretation more complex as we are often comparing the log-likelihoods of different classes of models (e.g., high LL models vs monotonic models) without making direct comparisons to the additive discrete model. Thus, we prefer to keep this portion of the Results as-is.

172: If the fit of the additive discrete DFE is slightly worse than gamma, how do we know that any changes to h are not, in some sense, trying to make the discrete DFE closer to the better-fitting gamma? In general, it would be good to give a sense for what frequency range is sensitive to changes in h or some other way to inspect model fit aside from raw likelihoods. The supplemental figures give some sense but are difficult to interpret.

**Response 15**

The best-fitting discrete DFE where *h*=0.5 for all mutations (i.e. the additive discrete DFE) has a log-likelihood of -1452.97. Of the 4096 discrete models with different values of *h* that we examined, the best-fitting model has a log-likelihood of -1451.68, which is only a 1.28 log-likelihood improvement. In fact, many of the models in our candidate set (those within 4.74 log-likelihood units of the additive model), actually have a slightly worse fit than the additive model. As such, the discrete DFE with arbitrary *h* is not greatly improving the fit compared to the additive discrete DFE.

However, more generally, we agree that it is good to see how sensitive the discrete DFE is to changes of different values of *h* for different bins. Figure 3 of the revised manuscript shows the impact of changing *h* for each selection coefficient bin under a discrete DFE model. Each plot shows the change in log-likelihood (ΔLL) relative to a model where all bins are assumed to be additive (*h*=0.5; LL=-1452.97). In each case, the dominance coefficient for the specified bin of the DFE (shown in each panel) was changed to a more recessive value (shown on the x-axis) while holding all other bins to *h*=0.5. Note that the model fit changes minimally as *h* becomes more recessive with the exception of making the weakly or moderately deleterious bins recessive. Strongly deleterious mutations show a complex pattern, where a model of *h*=0.05 results in a slight improvement in fit compared to the additive case while a fully recessive model (*h*=0) fits worse.

184: Justify why allowing h to vary among different classes is more important than allowing h to vary generally.

**Response 16**

Although it's hard to say which of these two factors is generally more important, it seems true that the evolutionary genetics literature on dominance has focused to a much greater extent on investigating the relationship between *h* and *s* rather than understanding the extent to which *h* can vary for a given *s*. This is what motivates our focus on the possible presence of an *h-s* relationship in humans, something that has not yet been done to our knowledge. Nevertheless, we agree that understanding the implications of more general variation in *h* is important, and now include a simulation analysis to explore this topic. See **Response 8** for details.

Figure 1A: y-axis is dominated by h=0 in a way that makes most of it hard to read.
Figure 1B/D: It seems most likely the the DFE is changing in a way that makes the h*s

distribution relatively constant. It would be natural to show the implied h*s distribution here instead.

**Response 17**

We thank the reviewer for these comments. The likelihood values associated with Figure 1A are included in Table S3 for interested readers. Although we agree it is hard to see minor differences among the top performing models in the plot, the main point we are trying to make here is simply that highly recessive models have very poor fit. Thus, we feel it is appropriate to leave the plot as-is given that the likelihoods are included in the supplement.

In **Response 6** above, we outline our reasons for presenting results in terms of *h* and *s* separately. However, we now include *h*s* for each gamma model in Table S3. Indeed, for models within 1.92 LL units of the MLE, *h*s* is relatively constant at ~0.0034. We now comment on this in the main text on lines 181-183:

*Indeed, we find that h*s is generally around ~0.0034 for gamma models with good fit, though increases to ~0.0045 for highly recessive models where the fit to the SFS was poor (**Table S3; Fig S1**).*

Finally, we also now include average *h*s* values for the discrete DFEs in Figure S7.

We also conducted a more detailed comparison of *h*s* for the gamma and discrete models when assuming all mutations have *h*=0.5. As noted in Table S3, for the gamma DFE, *h*s* =0.0033. The average *h*s* for the discrete model when assuming *h*=0.5 is 0.0173. The difference in these values comes primarily from how strongly deleterious mutations influence the mean *h*s* in the gamma vs. discrete DFEs. When only considering mutations that are not strongly deleterious (*s*<0.01), the mean *h*s* values inferred from the DFEs are much closer to each other (gamma: 0.0007, discrete: 0.00098). Thus, both the gamma and discrete DFEs provide similar conclusions in the part of the parameter space with the greatest statistical power.

We now discuss this issue on lines 545-551 of the revised manuscript where we write, *"While the gamma and discrete DFEs appear to have different inferred mean h*s values when assuming h=0.5 (gamma: 0.0034 discrete: 0.0173; **Table S3**; **Figure S7**), this is due to how strongly deleterious mutations influence the mean h*s in the two models. When only considering mutations that are not strongly deleterious (|s|<0.01), the mean h*s values inferred from the two DFEs are much closer to each other (gamma: 0.0007, discrete: 0.00098). Thus, both the gamma and discrete DFEs provide similar conclusions in the part of the parameter space with the greatest statistical power."*

221: Can the authors make any statement about whether monotonic decay models provide a better fit generally than models without this property?

**Response 18**

We thank the reviewer for this helpful question. We now comment on this in the Results on lines 262-267:

*Finally, although such models with a monotonic decay are expected based on previous work [14,17,18,25], we find that these monotonic decay models on average do not fit any better than high LL models without a monotonic decay (mean LL for monotonic decay = -1453.64, mean LL for high LL without monotonic decay = -1453.46;* ***Fig. 2****). Thus, our analysis does not provide any statistical support for or against the presence of an h-s relationship.*

234: Can the model averaging approach be used to provide a range of plausible h values rather than point estimates? Ranges seem more natural to the idea of constraining model space.

**Response 19**

This is an interesting point to consider. We agree that ranges are much more natural to consider than specific point estimates. Indeed, that is what we show in Fig. 2. There is a space of 120 models with a monotonic decrease in *h* as mutations become more deleterious that fit the data well. We use three of the models from this set for the simulations in Figure 4. To enable readers to more fully explore and use the set of possible models for their own analyses, we provide the parameter estimates and the log-likelihoods of each of the 4096 models in a .csv file as Table S7.

Showing a range of *h* values from the model averaging is tricky, as the different values of *h* for the different bins are not necessarily independent of each other. The same goes for the proportions of mutations inferred to be in each bin of the discrete DFE. These proportions are not independent of each other. Thus, we believe that it is more correct to consider the ranges of models shown in Table S7, rather than put ranges on the model averages.

253: What is the justification for using forward simulations when such quantities can be calculated in the diffusion approach?

**Response 20**

Using simulations to model genetic load is quite standard in the field (e.g., Simons et al. 2014; Henn et al. 2016; Harris & Nielsen 2016; Kyriazis et al. 2023) and for us offers a flexible way to formulate relatively complex models, such as the models with varying *h* we now include in our revised manuscript (see **Response 8**). For many of these more complicated models, it is not

immediately obvious how to compute quantities such as genetic load and inbreeding load using the diffusion approximation, thus we believe simulations are appropriate for our aims.

Figure 4: Boxplots appear to show significant variation among simulation replicates and are largely overlapping for the AF and EU populations. If that is correct, the comparisons of point estimates around line 280 in the text is misleading. A statistic like the “probability B_AF>B_EU” over possible evolutionary histories might be more appropriate.

**Response 21**

We thank the reviewer for noting this issue. The reason for this high degree of variation was our approach of projecting simulation results from just two autosomes to an entire genome, a procedure that was done to minimize the computational burden of the simulations but led to higher variance. In our updated manuscript, we have increased the number of modeled autosomes to 11 (though decreased the number of replicates from 100 to 25), which has greatly lessened the degree of variation among replicates, as shown below:

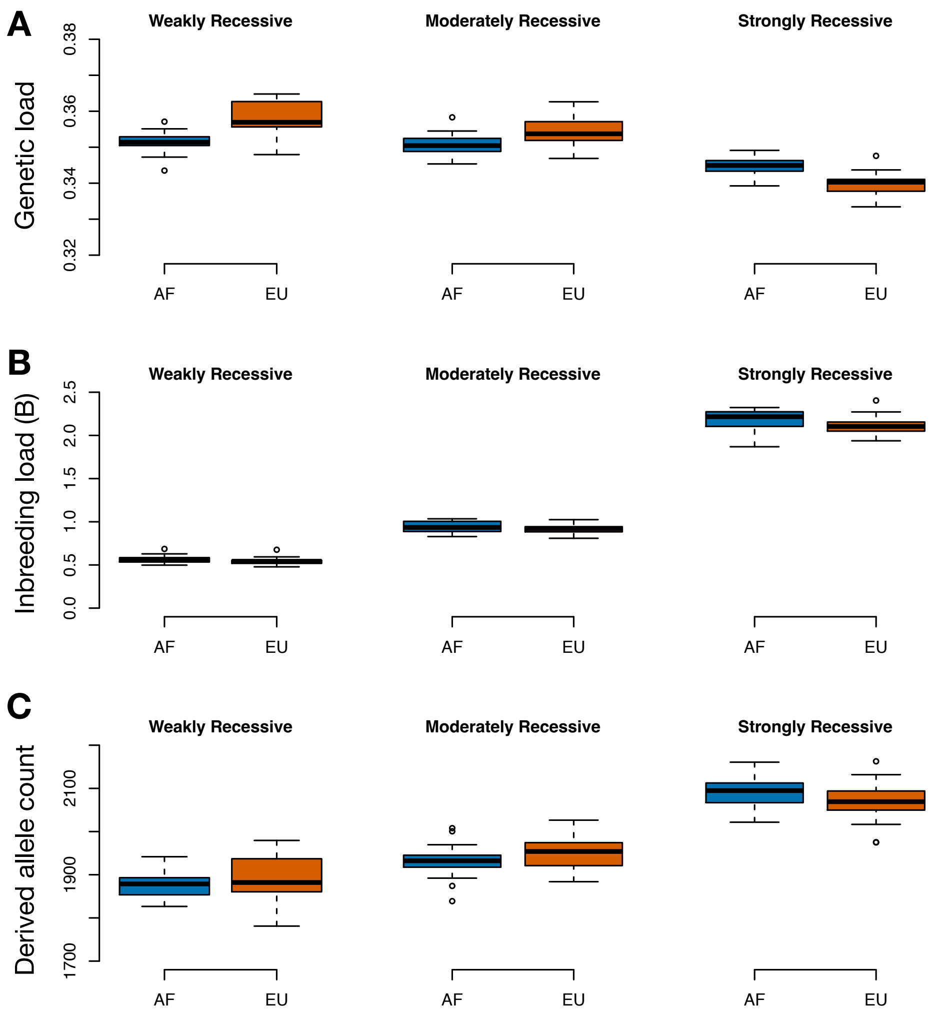

In light of these new results, we believe the comparisons made in the text are appropriate.

305: The authors should be clear about in what sense this h~0.35 is an average.

**Response 22**

We thank the reviewer for noting this confusion. We have now clarified in the main text that this average is for nonsynonymous mutations (on lines 360-362):

*Additionally, through model averaging, we estimate an average h for nonsynonymous mutations on the order of ~0.35, though with much greater uncertainty for h values of individual selection coefficient bins (**Fig. 2; Table 1**).*

**Reviewer #3:** This work attempts to disentangle the selection (s) and dominance (h) coefficient of a mutation, addressing an important and challenging aspect in genetics. It does so by fitting various models of selection and dominance to patterns of human genetic variation. The constrained models that best fit the data are then used to investigate if the Out-of-Africa bottleneck lead to differences in genetic and inbreeding load between Africans and non-Africans.

In their approach, the authors use the synonymous SFS to infer the demography and the nonsynonymous SFS to infer the DFE. Given that background selection might affect synonymous sites, the authors should justify their choice, compared to for instance inferring the demography based on the "neutral" part of the genome as defined in other studies (e.g., Pouyet et al., eLife, 2018). Replicating the results based on demographic inferences from a more reasonably "neutral" SFS would substantially improve this work. Alternatively, a cautionary note pointing to possible biases in the results emerging from biased demographic inferences should be provided.

**Response 23**

We thank the reviewer for this important comment. Indeed, we agree that background selection is pervasive across the human genome and its effects should be carefully considered in the context of DFE inference. Using synonymous variants to infer the demographic history actually helps control for the effects of background selection in DFE inference. The key idea is that, as the reviewer notes, the demographic models inferred from the synonymous variants may be biased by background selection. However, as synonymous and nonsynonymous mutations are interdigitated along the genomic sequence, they occur on the same coalescent genealogies. As such, the demographic model inferred from synonymous variants captures both the demographic effects as well as the linked selection effects. Inference of the nonsynonymous DFE will then be unbiased by background selection since it was accounted for in the demographic inference.

This idea was shown to apply in practice by Kim et al. 2017. Here, they used simulations to explore potential impacts of background selection on DFE inference using Fitðaði. They demonstrated that the correct DFE parameters could be estimated even in the presence of unmodeled linkage, though there were biases in the demographic parameter estimates. Of note, this is only an issue if one is interested in the demographic parameters themselves, rather than as treating demography as a nuisance parameter in the scheme of DFE inference.

We now comment on this issue as well as the potential impact of selection on synonymous mutations in the Methods on lines 474-479:

*Here, we assume that the synonymous SFS is neutral and that there is no linkage between synonymous and nonsynonymous sites, two assumptions that are likely violated in reality. However, Kim et al. [27] have previously shown that Fitðaði is able to infer the true DFE parameters despite the presence of unmodeled linkage, and Martinez i Zurita et al. [69] have*

*recently shown that small amounts of selection on synonymous variants does not impact DFE inference of nonsynonymous mutations*

The demographic inferences presented in this work are based on a pooled sample of Europeans. It is unclear if population structure within this sample can lead to the inference of parameters that are not suitable for human populations and how that could impact the results and conclusions. Does the range of dominance and selection coefficients hold for single populations?

**Response 24**

We thank the reviewer commenting on this issue, which was also noted by other reviewers. Although there is undoubtedly population structure in this European sample from the 1000 Genomes Project, previous analysis in Kim et al. 2017 demonstrated that such population structure does not impact inference of the DFE so long as the demographic model yields a good fit to the synonymous SFS. Additionally, Kim et al. 2017 also showed that the DFE inferred using the European sample from the 1000 Genomes Project was similar to the DFEs inferred from a more homogenous sample from Denmark as well as from the NHLBIO GO Exome Sequencing Project (ESP) data, which consists of a heterogenous sample of individuals with European ancestry. We now comment on this issue in more detail in the Methods (on lines 450-460):

*This European sample includes individuals from five different European populations (Utah residents with Northern and Western European ancestry, British in England and Scotland, Finnish, Iberian populations in Spain, and Toscani in Italy), among which there is pronounced population structure [38] that could potentially impact DFE inference. However, previous simulation analysis by Kim et al. [27] demonstrated that such cryptic population structure does not impact the performance of Fit∂a∂i, so long as the demographic model can fit the synonymous SFS. Additionally, this paper also showed that the DFE inferred using the European sample from the 1000 Genomes Project was similar to the DFEs inferred from a more homogenous sample from Denmark as well as from the NHLBIO GO Exome Sequencing Project (ESP) data, which consists of a heterogenous sample of individuals with European ancestry [27].*

Could the rejection of highly recessive models ($h<0.05$) be due to the restriction in the upper range of s? Can the authors provide an intuition for why lower values are not fitting the data? The finding of a lower bound of h for strongly deleterious mutations should be further discussed in light of previous findings.

**Response 25**

We thank the reviewer for noting this important issue of the lower bound on $h$ for strongly deleterious mutations. In our discrete DFE inference procedure, the strongly deleterious bin ranges in $|s|$ from 0.01 to 0.5, where 0.5 represents a lethal mutation when parameterizing the homozygote fitness as $1-2s$. Thus, there is no upper bound on $s$ in terms of the discrete DFE bins.

However, it is true that the grid of expected SFSs that we generated for Fit∂a∂i inference extend only to *s*=0.25. The implicit assumption in doing so is that we do not expect any mutations with |*s*|>0.25 to be segregating in our data. On a practical level, this assumption helps avoid some issues with numerical instability for the diffusion approximation with very strong selection. More broadly, this highlights the fact that a diffusion-based approach is may not be ideal for inferring *h* values for strongly selected mutations. To better highlight these limitations in our manuscript, we have added the following text in the Discussion (on lines 407-418, new text in bold)

*Instead, we find that an h for strongly deleterious mutations on the order of ~0.05-0.15 could better explain empirical estimates of the inbreeding load (**Fig. 4**). However, we note that a major limitation of our study is that we are unable to obtain fine-scale estimates of selection and dominance parameters for strongly deleterious mutations, which as defined here encompass a wide range of |s| from 0.01 to 1. This limitation is due to SFS-based methods being underpowered for estimating the strongly deleterious tail of the DFE [49], due to the fact that such mutations tend not to be segregating in genetic variation datasets [53–55]. Moreover, our diffusion-based approach may also be limited in inferring dominance parameters for strongly deleterious mutations given that the diffusion approximation breaks down under strong selection [56]. Given these considerations, our finding of a lower bound of h=0.05 for strongly deleterious mutations should be interpreted with some caution. Future work should focus on further refining selection and dominance parameters for strongly deleterious mutations.*

Finally, we emphasize that the lower bound is for the average *h.* However, if *h* is allowed to vary for each bin around this average, this allows for the presence of fully recessive mutations. We now explore this with a new set of simulations with variable *h* for each bin of *s* where we show that modest variation in *h* does not appear to impact the SFS. This implies that, if *h* truly does vary for mutations with the same *s*, we probably cannot detect this variability based on the SFS alone. See **Response 8** for more details on these new simulations. Thus, our estimates are consistent with there being some fully recessive mutations.

The results from Figure 3, show that the model fit is only slightly improved when h=0.05 relative to the fully additive model. This seems to point to a general lack of power to distinguish models (also seen in Figure 2 by the large range of models having a log-likelihood<1.92 units from that of the MLE). Predictive simulations to measure the power of this approach to disentangle h and s would be helpful to understand the robustness of the results.

**Response 26**

We thank the reviewer for this comment. We fully agree that power is expected to be limited when comparing various *h* and *s* models, an issue that is well established in the literature (e.g., see Fuller et al. 2019) and we do not claim to fully overcome this limitation in our analysis.

However, we agree that testing our inference approach on simulated data is a valuable component that was missing from our initial draft.

We have now carried out this analysis, which is presented in detail in **Response 7** above. To summarize, we find that the 'true' model was identified as having ΔLL=2.54 units from the MLE when fitting the full set of 4096 dominance models to the simulated nonsynonymous SFS. Moreover, when restricting to high LL models with a monotonic decay ($n$=55), we find that all models are quite similar to the true model, with weakly/moderately deleterious mutations ranging from partially recessive to additive and strongly deleterious mutations being more highly recessive. Overall, this analysis further strengthens the robustness of our approach for identifying a range of plausible dominance and selection models.

Minor comments:

I have identified a number of sentences who either lack or have some extra words (e.g., line 134-135, 253-255, and 296-298). I recommend a careful inspection and correction of the text.

**Response 27**

We are very grateful to the reviewer for catching these errors, which have now been corrected.

Please specify which parameter is shown in the y-axis of the bottom panel of Figure S3.

**Response 28**

This figure shows the proportion of mutations in each bin of the discrete DFE, which is labeled on the plot and in the figure caption. We have added an additional sentence at the end of the caption to further clarify this point.

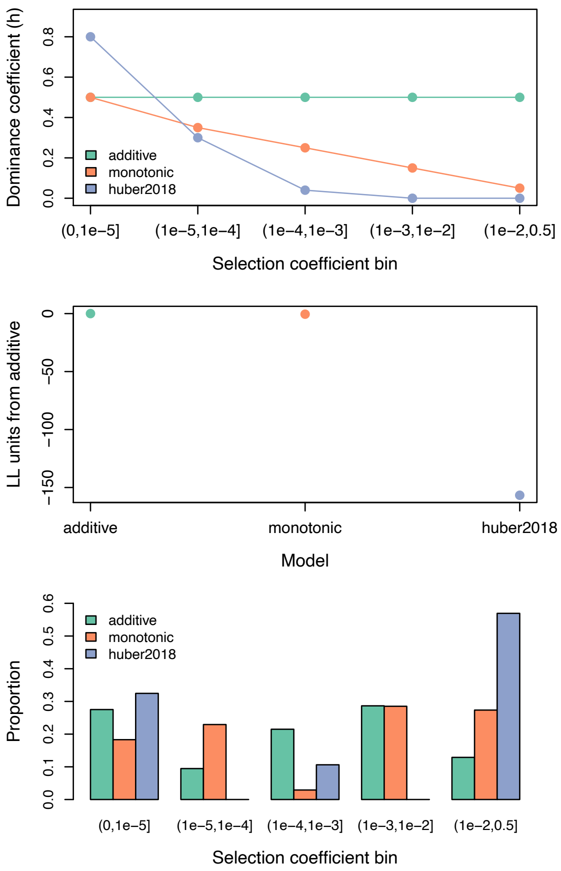


**Figure S3: The relationship between *h* and *s* inferred from *Arabidopsis* does not fit the human nonsynonymous SFS.** Top: Comparison of dominance parameters for models considered, including a fully additive model, a model with a monotonic decay in *h* that is within 1.92 LL units of the MLE, and the model from Huber et al. 2018 estimated for *Arabidopsis*. Middle: Model fit in comparison to the additive model. Note that the additive model and monotonic models have similar log-likelihoods though the Huber et al. 2018 model has a much worse log-likelihood. Bottom: Discrete DFE parameters estimated for each dominance model when fit to the human nonsynonymous SFS. Y-axis shows the proportion of mutations in each bin of the discrete DFE.

**Have all data underlying the figures and results presented in the manuscript been provided?**
Large-scale datasets should be made available via a public repository as described in the *PLOS Genetics* data availability policy, and numerical data that underlies graphs or summary statistics should be provided in spreadsheet form as supporting information.
Reviewer #1: Yes
Reviewer #2: Yes
Reviewer #3: **No:** The SFS for the 1000G project data is shown in Figures S1 and S2 and could be provided in spreadsheet form

We now include the 1000G synonymous and nonsynonymous SFS as Tables S5 and S6, respectively.
